# Antidepressant and Anxiolytic Potentials of the Chewing Stick, *Salvadora persica*

**DOI:** 10.1155/2023/9683240

**Published:** 2023-04-15

**Authors:** Rajalakshimi Vasudevan, Geetha Kandasamy, Afaf Aldahish, Mona Almanasef, Moteb Khobrani, Eman Shorog, Kousalya Prabahar, Enas Mohammed Alsawaq, Shadma Wahab, M. Yasmin Begum, Premalatha Paulsamy

**Affiliations:** ^1^Department of Pharmacology and Toxicology, College of Pharmacy, King Khalid University, Abha, Saudi Arabia; ^2^Department of Clinical Pharmacy, College of Pharmacy, King Khalid University, Abha, Saudi Arabia; ^3^Department of Pharmacy Practice, Faculty of Pharmacy, University of Tabuk, Tabuk, Saudi Arabia; ^4^Department of Pharmacy Practice, Faculty of Pharmacy, Dr. M.G.R Educational and Research Institute, Velappanchavadi, Chennai, Tamil Nadu, India; ^5^Department of Pharmacognosy, College of Pharmacy, King Khalid University, Saudi Arabia; ^6^Department of Pharmaceutics, College of Pharmacy, King Khalid University, Abha, Saudi Arabia; ^7^College of Nursing, King Khalid University, Abha, Saudi Arabia

## Abstract

**Materials and Methods:**

*Salvadora persica* stem bark was extracted with two different solvents, i.e., ethyl acetate and water, and preliminary phytochemical screening was performed. Two behavioral models were used: an elevated plus maze test (EPM) and the light and dark model test for anxiolytic parameters, and a forced swim test (FST) for antidepressant effects. Healthy mice weighing 18−40 gms were treated orally in four groups (*n* = 6), i.e., negative control treated with normal saline and positive control with 1 mg/kg diazepam (EPM) and 30 mg/kg fluoxetine (FST), and the test groups were treated with 500 mg/kg of aqueous and ethyl acetate Sp extract. The number of entries and duration spent in the open arm for 5 minutes were the parameters for evaluating the anxiolytic activity (EPM). Duration of immobility was measured for 5 min in the FST model.

**Results:**

In EPM, both the Sp extracts significantly (*p* < 0.005) increased the number of entries and the time spent in the open arms and was much similar to those of diazepam. Similarly, these extracts and fluoxetine significantly (*p* < 0.005) decreased the immobility time in FST.

**Conclusion:**

The results suggest the therapeutic potential of *Salvadora persica* an alternative in the management of comorbid anxiety and depression.

## 1. Introduction

Anxiety and depression are among the major psychiatric illnesses that affect individuals that cause disability and premature death [1]. By causing severe distress with increased suicidal beliefs, it typically prevails at any age, creating a tremendous burden for society. The underlying causal factors associated with these disorders include delayed medical symptoms, excessive stress, agony, medical history, socioeconomic burden, lack of family support, brain injury, and trauma [2].

World Health Organization approximates that 3.6% of people globally suffer from an anxiety disorder, and 4.4% suffer from depression, which is expected to be the leading cause of disability worldwide by 2017 [3]. Several studies in Saudi Arabia have predicted the incidence of such cognitive diseases, with rates varying between different populations, age groups, times, and geographic locations [4]. The systematic evaluation carried out by Ibrahim showed a general prevalence of psychiatric diseases of approximately 41%. To be precise, a study in the Asir region showed that the incidence of depression and anxiety was 27% and 25%, respectively [5, 6]. Previous studies have reported that almost 80–90% of people with depression report anxiety symptoms [7]. Diagnosing and treatment regimens become even more challenging due to overlapping comorbid conditions [8]. Although the patients responded to current treatments, the extent of improvement is still disappointing, coupled with the various physiological effects and tolerance on chronic therapy.

Current pharmacological interventions used in the management of these illnesses, unfortunately, often have numerous and severe side effects, including drug interactions, delayed response, and cases of nonresponse to treatment, among others [9]. Research on medicinal plants has continued to advance in the search for new molecules useful for the treatment of neurological disorders, showing the pharmacological efficacy of different plant species in various animal models. The search for psychoactive plants possessing therapeutic potential to treat anxiety and depression has attracted growing interest [10].

*Salvadora persica* (Sp), belonging to the Salvadoraceae family, is a medically significant species, also known as miswak and toothbrush tree, predominantly distributed in tropical and subtropical Asia. The plant has more medicinal value among the global Muslim community. Qualitative phytochemical studies on *Salvadora persica* showed the presence of alkaloids (salvadorine), new indole alkaloids (salvadoricine), glycosides, flavonoids, steroids, and saponins. Most of the published research works concentrated on its impact on oral health, showing its mechanical characteristics in plaque removal. Various ingredients of *Salvadora persica* have promising biological properties, including antimicrobial, antiallergic, antioxidant, anti-inflammatory, and antiproliferative activity. However, until now, very few scientific works have been reported on its antidepressant, and no research has accounted for its anxiolytic action. Also, the *Salvadora persica* exhibited virtually no toxic effects, no mortality, no overt evidence of delayed damage during the acute toxicity test, and no discernible impact on most biochemical and haematological measures [11–14]. Experimental paradigms such as elevated plus maze and forced swimming tests are widely used for identifying putative candidates for new treatment obtained from natural sources for anxiety and depression. Based on this information, this study aimed to evaluate the possible antidepressant and anxiolytic-like effects of *Salvadora persica* using behavioral models mentioned previously.

## 2. Materials and Methods

### 2.1. Plant Material

**Figure 1:**
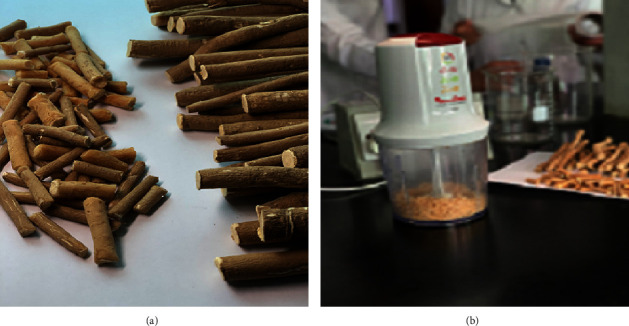
(a) Stem bark of *Salvadora persica* (miswak) and (b) miswak pulverised in a mechanical grinder.

The dried stem bark of *Salvadora persica* (Sp) (Figure 1) was obtained from the local market, Abha, Kingdom of Saudi Arabia, and was taxonomically identified by the Department of Pharmacognosy of the College of Pharmacy and Kingdom of Saudi Arabia.

### 2.2. Preparation of Aqueous and Ethyl Acetate Extract

The extraction of the *Salvadora persica* was performed using the maceration technique. First, the stem bark of *Salvadora persica* was dried and made into a coarse powder using a mechanical grinder (Figure 1). This dried stem bark powder (150 g) was dissolved in 750 ml of distilled water and ethyl acetate solvent in a separate glass bottles, mixed often, and kept in the dark, well-sealed glass bottles for up to 7 days at room temperature (35–37°C). The plant macerates were then filtered through Whatman No. 1 filter paper. Later on, both the extracts were evaporated to dryness at 45°C, in a rotary evaporator (Buchi Rotavapor). The final aqueous and ethyl acetate extracts of Sp were weighed. The extraction yield of aqueous and ethyl acetate extract were 16.89% and 5.08%. For biological evaluation of anxiety and depression model, we have taken 500 mg/kg of aqueous and ethyl acetate Sp extracts.

### 2.3. Preliminary Phytochemical Analysis

A preliminary phytochemical screening of the Sp extract was performed to identify the presence of secondary metabolites [15, 16]. For the detection of alkaloids (Dragendorff's test), saponins (foam test), tannins (ferric chloride test), and flavonoids (concentrated HCl test), qualitative analysis of phytochemical tests was carried out.

### 2.4. Animals

Healthy mice (*n* = 48) weighing 20–25 grams were selected for the study and were kept at ambient temperature (24 ± 1°C). The animals were allowed to have free access to standard pellets and water. The animals were obtained from the Animal House facility of the College of Pharmacy (King Khalid University). The study protocol is approved by the Ethical Committee of Scientific Research (ECM#2019−32), King Khalid University.

### 2.5. Behavioral Paradigms

#### 2.5.1. Anxiety Model

*(1) Elevated Plus Maze Assay*. The elevated plus maze test (Ugo Basile 108513, Italy) was performed according to methods described by to Pawlak et al. [17]. This experiment was commonly validated to measure rodent anxiolytic and anxiogenic activity. This apparatus, elevated from the floor, comprises two open arms and two closed arms, which have been extended from a central platform. In general, the mice were selected and classified into groups, as mentioned as follows:

  Group I (control): mice (*n* = 6) received normal saline orally (NaCl, 0.9% 10 ml/kg)
  Group II (standard): mice (*n* = 6) received an oral injection of 1 mg/kg [18]
  Group III (test): mice (*n* = 6) received 500 mg/kg of aqueous Sp extract orally [19]
  Group IV (test): mice (*n* = 6) received 500 mg/kg of Sp ethyl acetate extract orally [20]

The mice were placed separately in the maze's centre, head facing towards the open arm. The number of entries in the open and closed arms and the total time spent in open and closed arms, respectively, were recorded for 5 min (entry into an arm is described as the animal placing all four paws onto the arm). The observations were recorded for each animal, and the results were examined.

*(2) Light and Dark Model Assay*. The light and dark exploration (Ugo Basile 137713, Italy) test was described by Crawley and Goodwin in 1980 as a simple behavior model to detect the anxiolytic action of medicinal plants, steroids, and other compounds [21]. The light and dark model equipment consisted of a wooden box above. There are two separate compartments, a small compartment finished in black and also lined with black plywood over its top. While a bright room, painted white and brightly lit with the source of white light, was held over the open box. The two connected were joined via a small open doorway at the centre of the partition on the floor level; after 60 min of oral treatments, the mice were placed individually in the centre of the light box and observed for 5 min. Like the EPM model, mice were categorized into four groups: normal saline, diazepam, aqueous extract, and ethyl acetate extract. All the animals were treated orally.

#### 2.5.2. Depression Model

*(1) Forced Swimming Test (FST)*. The method was carried out on mice according to the method of Porsolt et al. [22]. This is the most widely used and recognized model for antidepressant activity assessment. The apparatus comprises a transparent plexiglass cylinder filled with water. Twenty-four hours before the actual swimming test, every animal was placed separately in the cylinder for about 15 min. For the first time, mice placed in the cylinder are initially extremely active, swimming vigorously in circles, attempting to climb the wall, or dive downward. After 2 minutes, activity begins to subside, mostly combined with immobility or increased floating time in the water. Before returning to their home cages, the animals were removed from the cylinder and allowed to dry in a warm enclosure. After 24 hours, they are placed again in the cylinder, and the immobility period (total duration in the immobility phase) is evaluated during the 5 min experiment. An animal is regarded to be immobile when it stays passively floating in the water and its nose found just above the surface along with a slightly hunched but upright position. About 30 minutes before the test, the test and the standard drug were administered as mentioned as follows:

  Group I (control): mice (*n* = 6) received normal saline orally (NaCl, 0.9% 10 ml/kg)
  Group II (standard): mice (*n* = 6) received an oral injection of fluoxetine 30 mg/kg [23]
  Group III (test): mice (*n* = 6) received 500 mg/kg of aqueous Sp extract orally [19]
  Group IV (test): mice (*n* = 6) received 500 mg/kg of Sp ethyl acetate extract orally [20]

#### 2.5.3. Statistical Analysis

All results were expressed as mean ± SEM (standard error of the mean). Statistical analysis (SPSS V.21) was performed using a one-way analysis of variance (ANOVA), followed by a post hoc Tukey test.

## 3. Results

### 3.1. Results of Preliminary Phytochemical Analysis

The presence of various phytochemicals, such as flavonoids, alkaloids, and tannin compounds, in both aqueous and ethyl acetate extracts of *Salvadora persica* isshown by qualitative phytochemical analysis tests in Table 1.

### 3.2. Results of Behavioral Studies

#### 3.2.1. Effect of Sp Extracts on the EPM Model

**Figure 2:**
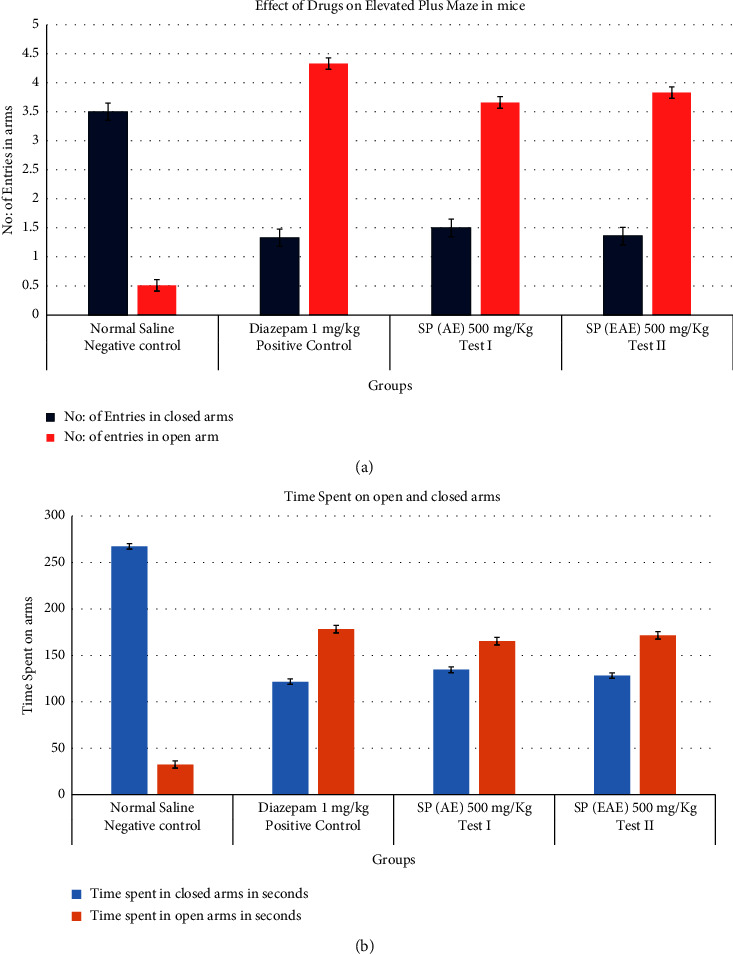
(a) Anxiolytic effect produced by different groups upon the number of entries in open and closed arms and (b) anxiolytic effect produced by different groups upon the time spent in open and closed arms.

As shown in Table 2, the oral administration of *Salvadora persica* aqueous extract and ethyl acetate extract at 500 mg/kg exhibited significant anxiolytic effects determined by an increase in the number of entries in the open arm (3.66 ± 0.2, 3.83 ± 0.12) compared to the negative control group (0.5 ± 0.34) and in a similar fashion that produced by diazepam (1 mg/kg) as shown in Figure 2(a). Additionally, these extracts significantly increased the time spent in the open arms (Figure 2(b)). Compared to the control group, extract treatment also showed a reduction in time spent and the number of entries in closed arms.

#### 3.2.2. Effect of Sp Extracts in FST Model (Table 3)

**Figure 3:**
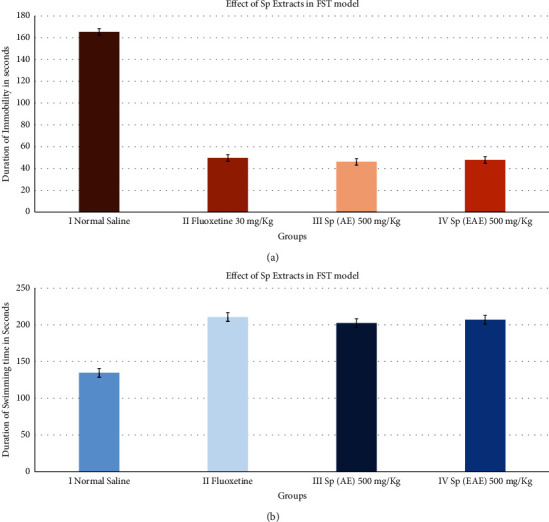
Antidepressant-like efficacy of treatment with Sp extracts in depressive behavior in response to FST. Immobility (a) and swimming (b) were recorded during FST. Columns show the means ± SD (*n* = 6).

The possible antidepressant effect was studied using the forced swim test. Administration of aqueous extract and ethyl acetate extract at 500 mg/kg of *S. persica* stem bark in mice demonstrated a significant reduction in immobility time when the animals were subjected to the forced swim test (Figure 3(a)). These extracts also significantly increased the swimming time compared to the control (Figure 3(b)). The results of fluoxetine (30 mg/kg) and the standard antidepressant were similar to those observed with the aqueous extract and the ethyl acetate extract.

Diazepam therapy significantly increased the amount of time spent (*p* < 0.001) in the light box and also the number of crossings between the light and the dark boxes, while in the dark box, the duration of time spent and the period of immobility (*p* < 0.001) were significantly reduced as shown in Table 4. Similarly, both *S. persica* extracts, when treated orally and in animals, also showed a significant increase (*p* < 0.001) in the time spent in the light box and the number of crossings compared to the vehicle-treated group. In addition, the duration of time spent and immobility period were significantly reduced in the drug-treated group.

## 4. Discussion

Globally, depression and anxiety are recognized as significant health problems among psychiatric disorders [24]. The quest for effective therapeutic agents that can produce antidepressant or anxiolytic effects with fewer side effects has evolved into growing interest. In neurobehavioral studies, herbal plants are essential for the development of potential therapeutic agents [25]. *Salvadora persica*, belonging to the Salvadoraceae family, is a commonly available plant in the Middle East region; despite its direct impact on oral health, very few works have been accounted for its neurobehavioral effect [13]. Based on this information, stem bark extracts have been studied for their anxiolytic and antidepressant activity.

The pathogenesis of multiple diseases, including psychiatric disorders such as depression and anxiety, has been associated with oxidative stress. Decreased plasma concentrations of antioxidants may be related to developing such psychiatric diseases [26]. According to the results of our study, phytochemical screening of the ethyl acetate and aqueous extract of *Salvadora persica* showed the presence of alkaloids, tannins, and flavonoids. Studies have demonstrated that phytochemicals such as alkaloids, flavonoids, and saponins possess anxiolytic effects in many animal models of anxiety [27]. Some studies have reported that flavonoids and alkaloids can act as defensive agents by inhibiting free radicals due to their antioxidant properties, thus enhancing favourable effects such as anxiolytic and antidepressant actions [28]. Therefore, the content of flavonoids and alkaloids and the other phytochemical compounds belonging to various phytochemical classes could have been involved in the biological response observed in this work.

Implementing animal models is essential for understanding the neurobiological basis of mood disorders and encourages the discovery of new therapeutic agents [29]. The forced swim test (FST) is used as a model to check depressed conditions in rodents by displaying animal hopelessness and mood. At the same time, elevated plus maze (EPM) is a commonly used rodent behavioral assay and has been validated to determine the impact of pharmacological agents on antianxiety [30].

In the EPM experiment, the normal saline-treated groups made fewer entries. They spent less time in the open arms than in the closed arms, according to the proven results that typically prevent the mice from entering the unprotected field. Conversely, pretreatment with the ethyl acetate and aqueous extracts of *Salvadora persica* showed an apparent anxiolytic-like effect. Sp extracts at 500 mg/kg produced an increase in open arms entries, as well as in the duration of time spent on open arms (i.e., anxiolytic-like action) in a manner similar to that of the standard drug diazepam (1 mg/kg). Additionally, Sp extracts also decreased the number of entries in enclosed arms compared to the control groups. In a similar study by Rabbani [31], *S. persica* administration resulted in a dose-dependent reversal of the anxiety-related parameters. Furthermore, compared to the cigarette smoke group, administration of *S. persica* extract significantly (*p* < 0.05) increased the amount of time spent in the open arm and the number of entries to the open arm at doses of 100 mg/kg and 200 mg/kg. Similar results were found in a study by the date palm and miswak aqueous extracts, which had antidepressant-like effects on depression-like behaviors in the model in rats and significantly (*p* < 0.05) increased the number of entries in the open arm [32]. According to a study by Biradar [33], utilizing the EPM model, Sp extract's administration significantly reduced transfer latency (TL). Thus, administration of Sp generated an anxiolytic-like effect that was reflected by a decrease in depressive-like behavior, which is consistent with other research studies that were conducted [31–34]. Thus, it is proving that the anxiolytic drugs exhibit improved performance in open arm exploration and the anxiogenic compounds work opposite by decreasing the entries to the open arm in the plus maze [35].

The light and dark exploration test was described by Crawley and Goodwin in 1980 as a simple behavior model to detect the anxiolytic action of medicinal plants, steroids, and other compounds [21]. Light and dark model research is a conflict study focused on ethological approach avoidance and is sensitive to medications that cause anxiety. In this study, the transition parameters are highly dependent on locomotive operation, the number of transitions between the light and dark compartments, and the time spent on the light side are considered anxiety indices [23]. This is the first study with the *Salvadora persica* on the light and dark model anxiolytic activity. Mice treated with aqueous and ethyl acetate of *S. persica* showed an increase in the amount of time spent in the brightly illuminated compartment and increased the number of crossings between the light and dark compartments, suggesting the fact that the effect may be the main anxiolytic parameter. The observed anxiolytic activity of *S. persica* may be due to the agonistic effect on the GABA receptor complex or may also be due to the 5-HT1B receptor blocker or as a 5-HT1A receptor agonist [24, 25].

Anxiety and depression usually appear as comorbid states, and treatment of either could result in a better outcome of these comorbid conditions [36]. Although selective serotonin reuptake inhibitors remain the drug of choice, significant adverse effects such as sexual dysfunction and nervousness would affect the compliance state of the patients, resulting in a poor outcome [37]. Hence, the potential antidepressant effect of Sp was assessed using the forced swim test (FST), with the working principle that when animals are subjected to unavoidable stress, they undergo a period of despair (immobility) with an alternate escaping nature. The forced swimming test is based on the idea that when an animal is placed in a water-filled container, it will initially try to escape but gradually become immobile, which could be seen as a sign of behavioral despair. Because this test involves subjecting the animals to stress, which has been demonstrated to play a part in the likelihood of severe depression, it has been widely employed. This simple behavioral procedure has since become a useful test for screening novel antidepressants and continues to form the basis for primary screening of psychoactive compounds. Immobility time in the FST is an index for measuring antidepressant-like activity. Antidepressant drugs reduce immobility time and increase swimming behavior. Most antidepressants reduce the period of immobility, and thus the FST is usually employed in the screening models of potent antidepressant drugs [38]. In a similar study [39], imipramine and the aqueous extract of *S. persica* (5 mg/kg and 10 mg/kg) significantly reduced (*p* < 0.05) the length of immobility in the FST compared to the control group. However, compared to the control group, *S. persica* extract (*p* < 0.05) increased swimming time significantly, resulting in an antidepressant effect. Furthermore, according to a study by Rabbani [31], the dose of *S. persica* at 100 mg/kg resulted in a significant (*p* < 0.05) decrease in the period of immobility and an increase in the number of dives when compared to the control group. When Sp was evaluated at 200 mg/kg, these alterations were discovered to be further dramatically altered, resulting in Sp's ameliorative impact on cigarette smoke-induced anxiety and depression in rats. Similar outcomes to our investigation were observed in another study using date palm and miswak aqueous extracts, which showed antidepressant-like effects on depression-like behaviors in the rat model and significantly (*p* < 0.05) decreased the length of immobility [32]. The other study by Ramadan et al. demonstrated that treatment with Sp extract in rats decreased immobile time, with a maximum reduction of 32.4% when provided at a dose of 900 mg/kg. Without affecting climbing much, SP extracts also significantly lengthened the swimming duration (16.8%) [34]. Thus, the findings of our study is similar to other studies conducted and verified that acute administration of the *S. persica* produced antidepressant like effect which reflected by decreasing depressive-like behavior [31–34, 39].

The present study demonstrates the antidepressant effects of the aqueous and ethyl acetate extract of *Salvadora persica*. Similar to a study in rats by Ramdan et al. [34], Sp extracts showed antidepressant activity comparable to fluoxetine. Reductions in the duration of immobility, considered the primary predictor of antidepressant efficacy, with a related rise in active behaviors such as swimming, were usually noted. Previous reports suggest that all antidepressants decrease rodent immobility, while other nonantidepressant drugs fail to respond.

The role of dynamic behaviors during the FST is another crucial notion. Although the behavior usually presented in articles is immobility, it has also been shown that the other measures are also significant. In particular, antidepressants that increase serotonergic neurotransmission resulted in longer swimming durations, while those found to increase catecholaminergic neurotransmission resulted in longer struggle durations. This has been found primarily and may be the explanation for the exposure to fluoxetine (selective serotonin reuptake inhibitors) in our tests which has resulted in increased swimming time but not struggling behaviors [40, 41].

As compared with the other studies, our study findings with both aqueous and ethyl acetate Sp extract at 500 mg/kg caused a significant (*p* < 0.005) decrease in immobility time as well as an increase in swimming behavior. Other researchers who examined the potential of different plant extracts have come to similar conclusions [17, 29]. Therefore, this behavioral profile may indicate that an association with the serotonergic system may result from such an antidepressant effect of Sp.

With these experimental tests, the mechanism by which *Salvadora persica* extract exerts action cannot be elucidated. Therefore, it is necessary to perform more pharmacological and biochemical studies, which will allow us to define whether the effects termed here result from any secondary metabolites obtained.

## 5. Conclusions

The present study indicates that the aqueous and ethyl acetate fraction of the *Salvadora persica* stem bark has anxiolytic and antidepressant effects in mouse models. The study's results suggest the therapeutic potential of *Salvadora persica* as an alternative in the management of comorbid anxiety and depression. Future studies include assessing other neurobehavioral models such as suspension tests and also to find the total flavonoid and alkaloidal content required for acting. Additionally, neurochemical studies are necessary to elucidate any effect of monoamine systems on the treatment of clinical depression or GABA-ergic action on anxiety improvement.

## Figures and Tables

**Table 1:** Preliminary qualitative test of stem barks of *Salvadora persica* (Sp) extracts.

S. no.	Phytochemical constituents	Ethyl acetate extract of Sp	Aqueous extract of Sp
(1)	Flavonoids	+	+
(2)	Saponins	−	−
(3)	Tannins	+	+
(4)	Alkaloids	+	+
(5)	Steroids	−	−

+ indicates presence and − indicates absence.

**Table 2:** Effect of Sp extracts in the EPM model.

Groups	Treatment (drugs and dosage)	No. of entries in closed arms	No. of entries in open arms	Time spent in closed arms (in secs)	Time spent in open arms (in secs)
I	0.09% n/saline (p.o)	3.5 ± 0.22	0.5 ± 0.34	267.5 ± 9.54	32.5 ± 1.26
II	DZP, 1 mg/kg (p.o)	1.33 ± 0.24^*∗*^	4.33 ± 0.22^*∗*^	121.7 ± 7.93^*∗*^	178.3 ± 3.16^*∗*^
III	Sp (AE) 500 mg/kg	1.5 ± 0.32^*∗*^	3.66 ± 0.2^*∗*^	134.6 ± 3.46^*∗*^	165.3 ± 3.69^*∗*^
IV	Sp (EAE) 500 mg/kg	1.36 ± 0.2^*∗*^	3.83 ± 0.12^*∗*^	128.3 ± 3.68^*∗*^	171.6 ± 2.90^*∗*^

Each value represents mean ± SEM (*n* = 6). ^*∗*^*p* < 0.005, in comparison with control. n/saline, normal saline; DZP, diazepam; Sp (AE), aqueous extract of *Salvadora persica*; Sp (EAE), ethyl acetate extract of *Salvadora persica.*

**Table 3:** Effect of Sp extracts in the FST model.

Groups	Treatment (drugs and dosage)	Duration of the immobility period in seconds	Duration of swimming time in seconds
I	0.09% n/saline (p.o)	165.33 ± 3.57	134.66 ± 5.13
II	FLX, 30 mg/kg (p.o)	49.33 ± 1.49^*∗*^	210.66 ± 3.97^*∗*^
III	Sp (AE) 500 mg/kg	47.83 ± 2.7^*∗*^	202.33 ± 7.75^*∗*^
IV	Sp (EAE) 500 mg/kg	46.16 ± 3^*∗*^	206.83 ± 5.3^*∗*^

Each value represents mean ± SEM (*n* = 6). ^*∗*^*p* < 0.005, in comparison with the control. n/saline, normal saline; FLX, fluoxetine; Sp (AE), aqueous extract of *Salvadora persica*; Sp (EAE), ethyl acetate extract of *Salvadora persica.*

**Table 4:** Effect of Sp extracts on the light and dark model.

Groups	Treatment (drugs and dosage)	Time spent in the light box	Time spent in the dark box	No. of crossings
I	0.09% n/saline (p.o)	129.33 ± 13.57	170.67 ± 12.13	14.65 ± 3.62
II	DZP, 1 mg/kg (p.o)	201.32 ± 11.44^*∗*^	98.68 ± 8.97^*∗*^	28.24 ± 2.68^*∗*^
III	Sp (AE) 500 mg/kg	198.66 ± 12.73^*∗*^	101.34 ± 10.14^*∗*^	25.38 ± 2.24^*∗*^
IV	Sp (EAE) 500 mg/kg	212.6 ± 13.32^*∗*^	87.4 ± 9.32^*∗*^	26.92 ± 3.12^*∗*^

Each value represents the mean ± SEM (*n* = 6). ^*∗*^*p* < 0.001, in comparison with control; n/saline, normal saline; DZP, diazepam; *Sp* (AE), aqueous extract of *Salvadora persica*; Sp (EAE), ethyl acetate extract of *Salvadora persica.*

## Data Availability

The data supporting the current study are available from the corresponding author upon request.

## Additional Points

*Importance of the Work*. According to the current study, the *Salvadora persica* stem bark fraction has anxiolytic and antidepressant effects in mouse models. The study's findings point to *Salvadora persica*'s therapeutic potential as an alternative for treating comorbid anxiety and depression. Future research will examine different neurobehavioral models, such as the suspension test. To clarify any effects on monoamine systems in treating clinical depression or GABA-ergic action in the reduction of anxiety, neurochemical studies are required.
